# Observations on the pollination and breeding systems of two *Corybas* species (Diurideae; Orchidaceae) by fungus gnats (Mycetophilidae) in southwestern Yunnan, China

**DOI:** 10.1186/s12870-022-03816-1

**Published:** 2022-09-02

**Authors:** Zhou-Dong Han, You Wu, Peter Bernhardt, Hong Wang, Zong-Xin Ren

**Affiliations:** 1 CAS Key Laboratory for Plant Diversity and Biogeography of East Asia, Kunming Institute of Botany, Chinese Academy of Sciences, Kunming, China; 2 University of Chinese Academy of Sciences, Beijing, China; 3 The Missouri Botanical Garden, Saint Louis, MO USA; 4 Lijiang Forest Biodiversity National Observation and Research Station, Lijiang, China

**Keywords:** Fly pollination, Fungus mimicry, Deception, Pollinator, Cross-pollination

## Abstract

Modes of floral presentation in some angiosperms attract flies that eat and/or oviposit on seasonal fruiting bodies of fungi. Mushroom mimesis by orchid flowers has been speculated in the geoflorous, Indo-Malaysian-Australasian, genus *Corybas* s.l. for decades but most studies remain fragmentary and are often inconclusive. Here we report the roles of fungus gnats as pollinators of *Corybas geminigibbus* and *C. shanlinshiensis* in southwestern Yunnan, China, combining results of field observations, lab analyses, and manipulative experiments. Hand pollination experiments suggested both species were self-compatible but incapable of mechanical self-pollination, thereby requiring pollinators for fruit production. A female of a *Phthinia* sp. (Mycetophilidae) carried a pollinarium of *C. geminigibbus* dorsally on its thorax. Two females and one male of *Exechia* sp. (Mycetophilidae) visiting flowers of *C. shanlinshiensis* carried dorsal depositions of pollinaria on their thoraces. Mycetophilid eggs were not found in the flowers of either species. The comparative fragrance analyses of these flowers and three co-fruiting mushroom species did not suggest that either orchid species was a brood-site mimic. This is the first confirmation of the dispersal of pollinaria of *Corybas* species by fungus gnats in subtropical-temperate Asia.

**Supplementary Information:**

The online version contains supplementary material available at 10.1186/s12870-022-03816-1.

## Introduction

Explosive speciation within the family Orchidaceae Juss. appears to be driven by multiple factors including the evolution of labile and exaggerated pollination mechanisms [1]. This includes a pronounced trend towards pollination-by-deceit in which orchid flowers provide a predictable suite of floral attractants but fail to offer food rewards, receptive females or suitable brood sites [1, 2]. Specifically, pollination by members of the order Diptera have evolved independently in multiple lineages in the orchid family. Some fly-pollinated species offer food rewards. Some are mimetic and others combine rewards and mimicry [3–7]. One of the most interesting trends within fly-pollination in the Orchidaceae is exploitation of micro-dipterans. These insects have bodies less than 5 mm in length and belong to several unrelated families including the Ceratopogonidae, Chloropidae, Drosophilidae, and Mycetophilidae. Pollination by micro-dipterans has been reported most often in the subfamilies Orchidoideae and Epidendroideae especially within the species rich subtribe of Neotropical Pleurothallidinae Lindl. ex G. Don [8] and the Australian tribe Diurideae (Endl.) Lindl. ex Meisn. [5, 9, 10]. As pollinator diversity within these taxa has not been fully sampled yet, the role of micro-dipterans in orchid pollination may reflect an underestimated component within evolutionary ecology.

Among the lineages within the Orchidaceae pollinated by micro-dipterans, some show a trend towards fungus-gnat pollination. These flowers exploit flies 2–5 mm in length recorded previously as feeding and/or laying their eggs on complex fruiting bodies of fungi or their mycelia**.** However, the common name, fungus gnat or fungus fly, refers to species in at least seven families including the Drosophilidae, Sciaridae, Diadocidiidae, Ditomyiidae, Keroplatidae, Bolitophilidae and Mycetophilidae. Orchid species producing flowers indicative of a fungus-gnat syndrome are known for their fishy-fungus-like scents and sculptures on their labella purportedly resembling hymenophores, as in the genus *Dracula* [11, 12]. Other species bloom close to the ground and their perianth segments appear pouched or chamber-like [13, 14].

In southern Australia, several genera in the Diurideae including *Acianthus* R. Br., *Corybas* Salisb., *Pterostylis* R. Br. and their relatives [sensu 10] bloom during the wet, cool, winter-early spring when mushrooms appear. These orchids may form a guild exploiting males and/or females of gnats in the families Keroplatidae, Mycetophilidae and Sciaridae [15]. *Corybas* s.s. and its three related genera [sensu 10] comprise a lineage of approximately 130 species [3] in the tribe Diurideae. They are distributed broadly from the Himalaya south through Australasia with additional Indo-Malaysian and Papuasian centers of diversity [10]. Flowers in this lineage are geoflorous and resupinate with a dome-like dorsal sepal giving them the common name of helmet orchids. Most are dark (iodine) colored with sculpted labella suggesting they may mimic a small mushroom with a smooth, concave, pileus [13].

Pollination by fungus gnats in this lineage in Australia was first proposed by Jones [13] who found a dead mycetophilid female bearing a dorsally deposited pollinarium on its thorax in a flower of *C. diemenicus* (Lindl.) Rupp at Mount Morton, Victoria. Jones suggested the gnat entered the flower to feed from a pair of glands he found at the base of the column. Despite this initial study there have been few publications documenting the dispersal of *Corybas *s.l. pollinaria by fungus gnats. Kelly et al. [16] completed an exhaustive study on *C. cheesemanii* (Hook.f. ex Kirk) Kuntze in New Zealand and recorded a number of flies in different families visiting its flowers and co-fruiting basidiomycetes. They concluded that only males in the genus *Mycetophilia* could actually enter and exit the flowers but failed to find pollinaria on the bodies of gnats. *Mycetophilia* species bearing pollinia fragments of *C. iridescens* Irwin & Molloy in New Zealand were collected from flowers under cultivation and these visits appeared responsible for fruit production [17].

To date, the most convincing studies of fungus-gnat pollination in five *Corybas* species are based on photographs and descriptions by Kuiter [18] and additional observations in an independent, but non-peer reviewed publication by Kuiter and Findlater-Smith [19]. They found that the flowers of each species were specialized in two ways. First, they were either pollinated by mycetophilids in the genus *Phthinia* or *Mycetophilia*. Second, due to interspecific variations in dimensions of the floral sinuses coupled with intergeneric differences in insect sexual dimorphism, each *Corybas* species was pollinated either by male or female gnats. The authors noted that the insects lapped the labella with their proboscides and Kuiter (personal communication) insisted that labella of all five *Corybas* species secreted micro-droplets rejecting earlier interpretations that the flowers are brood site mimics [15].

As in most orchid genera, even less is known about the breeding or compatibility systems [1, 20] in the *Corybas* lineage. Some Australasian species undergo obligate self-pollination [10]. In contrast, Coleman [21] reported that up to 50% of the pollinaria in populations of *C. aconitiflorus* Salisb. (syn. *Corysanthes bicalarata* R. Br, sensu Jones et al. [10]) were removed by unknown insects. However, only 1% of its flowers showed evidence of pollen on the stigma and/or set fruit. The rate of fruit set varies from 23% in *C. ecarinatus* Anker & Seidenf. in Thailand [22] and 25% in *C. cheesemanii* in New Zealand [16]. A low rate of conversion of flowers into fruit is most often associated with orchids with mimetic flowers [1, 20] but it also constitutes an increasing concern for global conservation of orchid populations, in general. It is the time for additional analyses of pollination and reproductive success in Asian *Corybas* species.

The southwest mountains of China and the Himalaya are the northernmost distribution of *Corybas* s.s. with some species found at elevations up to 2000 m. The Gaoligong Mountain bordering China and Myanmar harbors four species, *Corybas himalaicu*s (King & Pantling) Schlechter, *C. shanlinshiensis* W. M. Lin, T. C. Hsu & T. P. Lin, *C. sinii* T. Tang et F. T. Wang, and *C. geminigibbus* J. J. Sm., the last species was a new record for China (Han et al., unpublished data). This mountain provided populations large enough to observe pollinators and perform hand-pollination experiments on *C. geminigibbus* and *C. shanlinshiensis* for one season. We addressed the following questions, 1) do both orchid species require pollinators for reproduction? 2) if either species requires a pollinator, which insects pollinate them? 3) Based on field observation and lab analyses, what are the attractants and rewards employed by these flowers? Our results offer additional insights into the puzzle of fungus gnat pollination *vs.* the theory of mushroom mimicry in the genus *Corybas.* It provides basic information on the reproductive cycles of two species that will probably require conservation management in the near future.

## Materials and methods

### Study species and sites

**Fig. 1:**
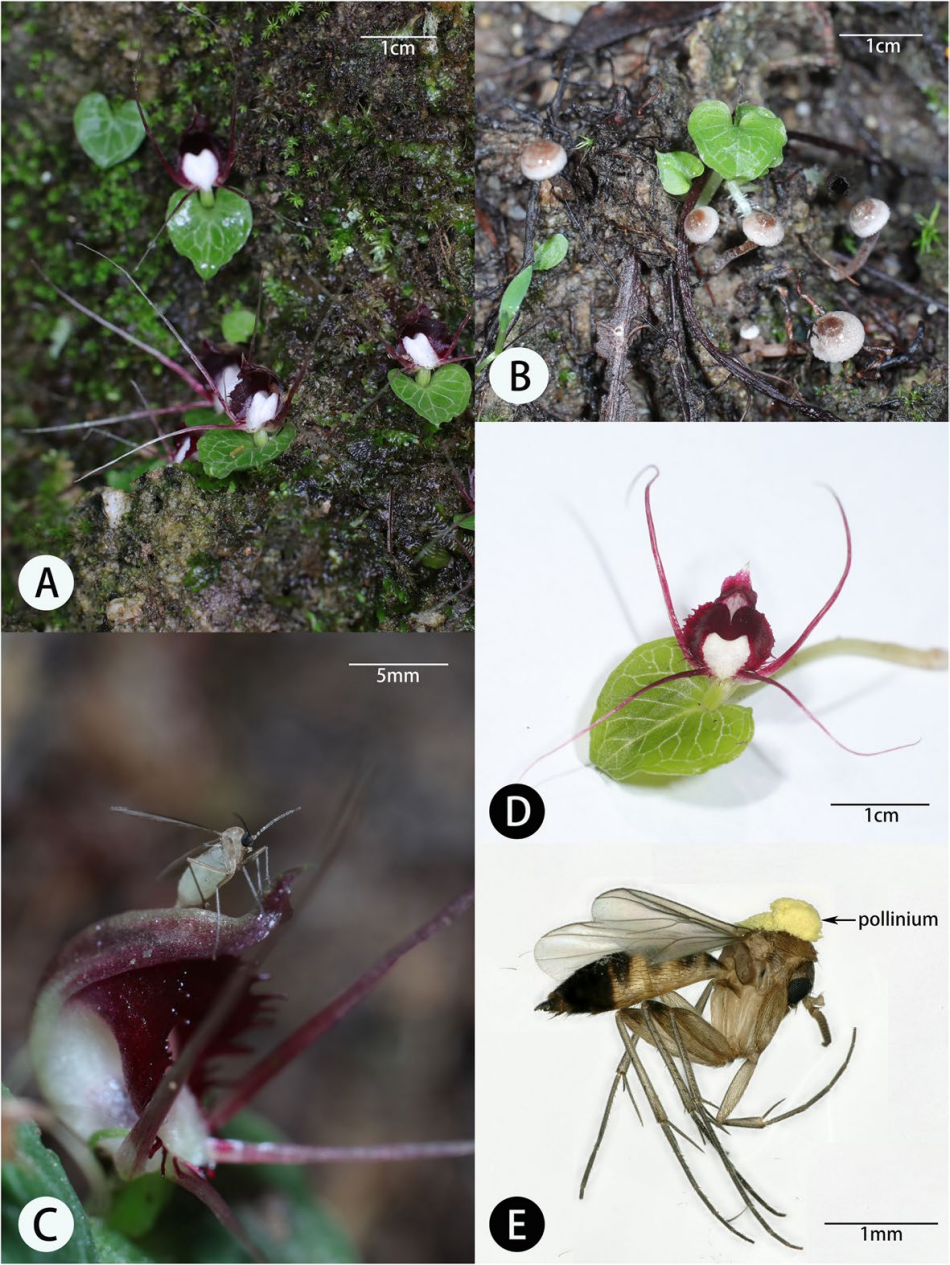
Habitat, flowers and pollinators of *C.* *geminigibbus* in southwestern Yunnan. A, Habitat, B, two non-flowering plants with several basidiocarps of *Psathyrella* sp. The flower of these orchids bloomed after mushroom fructification. C, Fungus gnat on the dorsal sepal of the orchid, D, flowering plant, (E) Female fungus gnat (*Phthinia* sp.) carrying a pollinarium on her thorax

*Corybas geminigibbus* is distributed in Thailand, Peninsular Malaysia, Indonesia and the Philippines. Its discovery in a lower montane forest in southern Thailand occurred in 2016 [23]. Flowers of this species are small with a single leaf and a single flower per leaf (Fig. 1A, D). The central part of the labellum is white with dark purple margins ending in a short spur. Our study populations in southwestern Yunnan represent an additional extension of its known distribution (Han et al., unpublished data). We reserved two sites for pollinator observations, one at Xiangbaihe (XBH) with an elevation of 1476 m, in Longling county, near Baoshan city. The second was at Mangbangzhen (MBZ) with an elevation of 1366 m, near Tengchong city. We do not provide GPS data for the sites due to the illegal trade in wild orchids. The vegetation of both sites was similar with a monsoon evergreen broad-leaved forest dominated by *Schima wallichii* (DC.) Korth. as the dominant tree. *Ardisia shweliensis* W. W. Smith grew extensively within the shrub-understory layer, and *Dicranopteris pedata* (Houttuyn) Nakaike was a common woodland herb. Both sites were highly fragmented due to human disturbance. The XBH population was located on a river bank bordered by crop fields with sections of the river banks destroyed by dredging. There were more than 300 flowering individuals at each site blooming July to August in 2020. Fruits matured in September.

*Corybas shanlinshiensis* is also a new record in Yunnan, found previously only in Shanlinshi, Nantou county, Taiwan [24]. It is also small and geoflorus producing only one flower per leaf but the flower is larger than that of *C. geminigibbus* (Table 1, Fig. 3A, B). The flower of *C. shanlinshiensis* is similar to that of *C. sinii* Tang & F. T. Wang but has a much narrower floral opening (sinus). The spur is about 4 mm long. The site for *C.* *shanlinshiensis* was located at Houqiaozhen (HQZ) at an elevation of 1795 m, in a *Pinus yunnanensis* Franch. and *Quercus aliena* Blume woodland with *Rhododendron delavayi* Franch. as the dominant shrub. There were about 150 flowering individuals growing on mossy and humus rich slopes in 2020. Flowering was observed from July to August. Fructification was complete by September.

**Table 1:** Morphometrics of *Corybas geminigibbus*, *C. shanlinshiensis* and their pollinators (mm)

	Number of flowers/pollinator	Mean	SD
*Corybas geminigibbus*			
Opening (sinus) width	10	2.35	0.38
Opening (sinus) depth	10	2.5	0.36
Spur length	10	1.56	0.15
Lateral petal length	10	21.65	2.90
Lateral sepal length	10	16.85	3.16
Depth from stigma to lip surface	10	1.38	0.18
Gnat length	1	2.13	NA
Gnat thorax depth	1	0.67	NA
*Corybas shanlinshiensis*			
Opening (sinus) width	16	4.02	0.73
Opening (sinus) depth	16	4.62	0.66
Spur length	16	3.88	0.49
Lateral petal length	16	13.83	1.69
Lateral sepal length	16	11.53	1.35
Depth from stigma to lip surface	16	2.18	0.16
Gnat length	3	4.08	0.31
Gnat thorax depth	3	1.41	0.02

Voucher specimens of both species (*C. geminigibbus*: HZD20060 from XBH and HZD20069 from MBZ; *C. shanlinshiensis*: HZD20013) were identified by ZDH, and deposited in the herbarium of the Kunming Institute of Botany (KUN), Chinese Academy of Sciences, Kunming, China. Field work conducted on Gaoligong Mountains and specimen collections were supported and permitted by the Special Foundation for National Science and Technology Basic Research Program of China (2021FY100200) following the rules of the Convention on the Trade in Endangered Species of Wild Fauna and Flora (https://www.cites.org/).

### Floral longevity, floral morphometrics and rewards

In 2020, we selected 15 flowers of each species for recording the day flower buds open until the day the perianth segments wilted. We defined a flower as open on the day when perianth segments expanded opening the floral sinus allowing insects to enter. The floral lifespan was regarded as finished when perianth segments collapsed closing the sinus denying further entry to insects. To measure floral morphometrics, we selected 10 open flowers (see above) of *C. geminigibbus*, and 15 flowers of *C. shanlinshiensis* randomly. We measured the following floral traits with digital calipers to an accuracy of 0.01 mm: 1) floral opening width, 2) floral opening depth, 3) spur length, 4) length of the left lateral petal, 5) the length of the left lateral sepal and 6) the depth from the stigma to the surface of the lip directly under it. We dissected additional, fresh flowers of each species under a dissecting microscope to look for sites of nectar/micro-droplet production and evidence of insect eggs (see below).

### Pollinator observation

Observations of insects visiting flowers occurred in situ from 8:00—18:00 in July and August in 2020. We observed insect activity in *C. geminigibbus* for 300 h (about 30 days), and 150 h (about 15 days) for *C. shanlinshiensis*. We recorded visitors to the orchid flowers and to sympatric mushrooms to see if any carried orchid pollinaria. Insects observed carrying pollinaria as they visited targeted flowers and/or fungi were netted and stored in separate centrifuge tubes. Specimens in tubes were carried back to the field lab and frozen at -20℃ for one hour. After thawing, they were photographed and we recorded the location and number of pollinaria attached to their bodies. Using digital calipers, we measured body length and thorax depth with a resolution of 0.01 mm [25]. Insect voucher specimens were photographed with a Canon 5D Mark4 camera, then pinned and sent to entomologists for identification. Specimens were deposited in the KIB.

As *Corybas* spp. are suspected of brood site mimicry, we checked all flowers of each species over their floral spans during their respective flowering periods at each site. We could not observe egg-laying when an insect entered the flower as when a fly enters the orchid through the sinus it is no longer visible. Therefore, when we observed flies on orchids we spread or probed floral segments to see if any insects were in the process of laying eggs.

### Mushroom observation and collection

We observed and collected mushrooms while *Corybas* species were in bloom. At each site we also checked the stipe, pileus, and hymenia of about 100 mushrooms with mature fruiting bodies for egg-laying insects and searched for previously laid eggs. We took the photos of each mushroom species, and specimens were collected and dried in a field oven. Photos and dried mushroom specimens were identified by mycologists at the KIB. Voucher specimens of mushrooms were also deposited in the herbarium of the Kunming Institute of Botany (KUN), Chinese Academy of Sciences, Kunming, China.

### Scent analyses of flowers and mushrooms

**Fig. 2:**
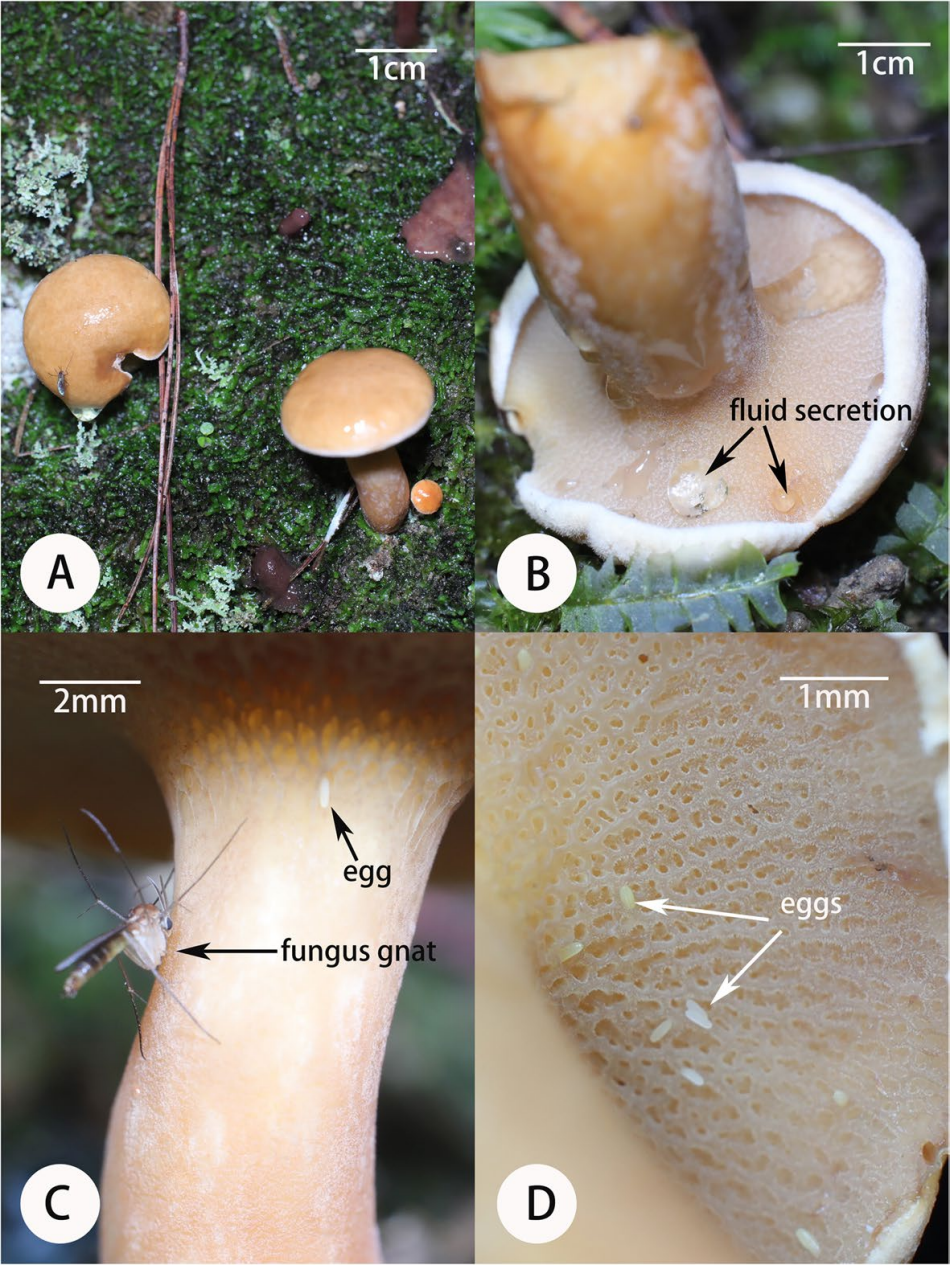
Mushrooms and fungus gnat activity on mushroom at the same site as the flowering *Corybas geminigibbus*. A, Basidiocarps of *Suillus bovinus*; B, Inverted mushroom (*S. bovinus*) showing hymenophore secretions. C. Unidentified fungus gnat on mushroom stipe and an egg on the same stipe; D. Unidentified eggs of fungus gnat on hymenophore surface

As the flowers of both *Corybas* species grew close to the ground, in wet habitats, and in the presence of basidiocarps that were easily trampled, we collected fresh flowers (opened for 3–5 days) for scent analysis to avoid environmental contamination. We conducted floral and mushroom scent collection in a field lab in MBZ and HQZ. We selected three sympatric mushrooms (*Suillus bovinus*, *Mycena* sp. and *Laccaria* sp.) for scent analysis, because they grew within a few centimeters of our orchids. *Suillus bovinus* and *Mycena* sp. occurred within the same habitat as *C. geminigibbus*, and we observed initial fungus gnat activity on basidiocarps of *S. bovinus* (Fig. 2). *Laccaria* sp. came from the same habitat as *C. shanlinshiensis*. Only fresh mushrooms were collected for scent collection. Floral/mushroom scents were collected by a dynamic headspace method as described by Tao et al. [26]. A headspace bag (Reynolds®Oven Bag; Reynolds, Inc., Richmond, VA, USA) was cut to dimensions of 10 × 10 cm and used to cover 6–12 flowers or six individuals of the same mushroom species per sample. An adsorbent trap tube (Tenax tube, GL Science, Tokyo, Japan) with 130 mg thermal desorption Tenax TA (Shimadzu Group Company, Kyoto, Japan) packed between glass wool was attached to a battery-operated PAS-500 vacuum pump (Spectrex Inc., California, USA) with Tygon tubing. The terminus of the trap was then sealed within the headspace bag with a twist tie. We cut a second hole in the bag for air flow. Floral or mushroom scent collection occurred over several two hour periods from 09:00 to 19:30 on 23–26 July, 2020 at a standardized flow rate of 60 ml air/min. Ambient air controls were included to account for non-floral/mushroom compounds. Trap tubes after scent collection were stored at -20 °C.

The volatiles were analyzed on a Nexis GC-2030 GC System coupled to a GCMS-QP2020 NX (Shimadzu Group Company, Kyoto, Japan) by a thermal desorption method using a TD30 Thermal Desorption system. Thermal desorption temperature was 250 °C held for 5 min. A RTX-5 column (5% Phenyl-Methylpolysiloxane; 30 m long; inner diameter 0.25 mm; film thickness 0.25 μm; Restek, USA) was used for analyses. Electronic flow control was used to maintain a constant helium gas flow of 1.0 ml/min. The GC oven temperature began at 40 °C and increased 3 °C per min to 80 °C, then it was increased to 5 °C per min to 280 °C and held for 5 min. The MS interface was set at 250 °C and the ion trap worked at 230 °C. The mass spectra were taken at 70 eV (in EI mode) with a scanning speed of one per scan from m/z 35 to 500. Component identification was carried out using NIST17 and FFNSC1.3 mass spectral database.

### Breeding systems

To test if either *Corybas* species depended on pollinators to deposit pollen on their stigmas we conducted four manipulated treatments in July and August 2020. To stabilize and protect each flower during bagging, a wooden skewer was inserted next to each plant and the bagged flower was attached to the skewer to hold it in place. As the flowers of both species were small, we had to carefully cut the lip into two parts to expose the stigma for hand pollination. Bags were replaced after each of the following manipulations. 1) Bagged control: flower buds were isolated under organza bags and tagged before perianth expansion and remained bagged for the flowering season (*N* = 15 for both species). 2) Hand self-pollination: flower buds were bagged and tagged but when the perianth segments opened we removed the friable pollinia in the solitary anther with forceps and smeared it onto the stigma of the same flower re-applying the bag for the remainder of the season (*N* = 10 for *C.* *geminigibbus*; *N* = 15 for *C. shanlinshiensis*). 3) Hand cross-pollination: bagged and tagged flowers had their pollinia removed, and then their stigmas were pollinated using pollen taken from an open flower blooming at least 2 m away (*N* = 10 for *C.* *geminigibbus*; *N* = 15 for *C. shanlinshiensis*). 4) Natural pollination: plants were tagged in bud but never bagged and exposed to resident insects throughout their floral life spans (*N* = 68 for *C.* *geminigibbus* in XBH; *N* = 110 for *C. shanlinshiensis*). We returned to our sites in the late September to check for fruit production in all four treatments.

## Statistics

For both species, differences in fruit production between pollination treatments were assessed using a Generalized Linear Model (GLM) with binomial errors distribution and a logit link function. Each pollination treatment was regarded as a fixed effect so fruit production was coded as “set no fruit” (0) and “set fruit” (1) as a binary response variable. We assessed significances of the GLM model with likelihood-ratio tests using the ANOVA function in R package car [27]. Post hoc multiple comparison tests using the glht function in multcomp package [28] were used to detect for differences between pollination treatments. We used a Mann–Whitney Rank Sum Test to compare flower dimensions of *C. shanlinshiensis* with the body sizes of its pollinators in order to test matching dimensions between flowers and insects.

## Results

### Floral phenology, longevity and morphometrics

**Fig. 3:**
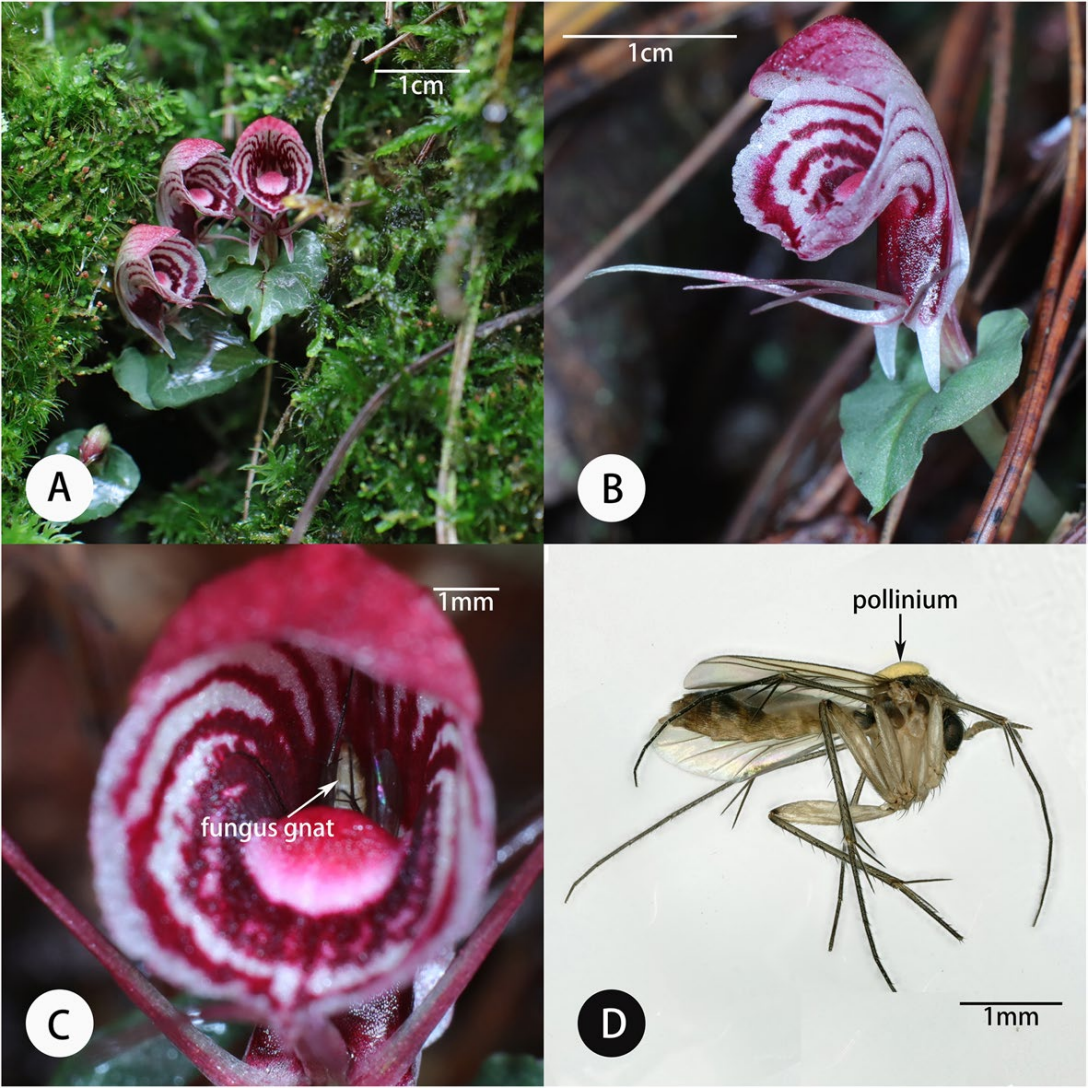
Habitat, plant and pollinator of *Corybas shanlinshiensis.* A. Flowering plants in a mossy and humus rich habitat; B. A flower; C. Abdomen of a fungus gnat after inside the floral sinus; D. A female fungus gnat (*Exechia* sp.) wearing a pollinium of *C. shanlinshiensis* on its thorax

The flowering season of both species overlapped from July to August during the rainy season. Individual flowers of *C. geminigibbus* opened and lasted 18.7 ± 1.5 days (Mean ± SD, *N* = 15), while floral longevity of *C. shanlinshiensis* was shorter, lasting 14.8 ± 3.5 days (*N* = 15). We did not detect nectar or any fluid secretions in whole or dissected flowers of either species. In both species, petals and lateral sepals are the most elongated floral organs (Fig. 1, 3). The flower of *C. geminigibbus* had an open sinus width and depth of less than 2.5 mm. The same measurement was smaller for *C. shanlinshiensis*. The opening width and depth of *C. shanlinshiensis* was less than 4.62 mm and the spur length was 3.88 ± 0.49 (*N* = 16; Table 1).

### Pollinators behavior and mushroom diversity

We did not find the eggs of fungus gnats in the flowers of either species. We observed fungus gnats visiting flowers of *C. geminigibbus* three times following 300 h of observation. The insects were hard to see with the naked eye as they approached the flowers (Fig. 1C). We were able to collect only one specimen carrying a solitary pollinarium on the dorsum of its thorax at site XBH. A second specimen, also bearing pollinia escaped from the net. Both collections occurred from 11:00 am to noon. The solitary specimen was identified as a female *Phthinia* species in the Mycetophilidae (Fig. 1E). Its thoracic depth was 0.67 mm, less than the distance between the stigma and the surface of the lip of *C. geminigibbus.*

Based on visible fruiting bodies, there were > 16 species of basidiomycetes in the *C. geminigibbus* sites (Table S1). Some species, including *Psathyrella* sp. emerged before the first flower buds of this orchid opened (Fig. 1B). *Suillus bovinus* (Fig. 2A), *Strobilomyces echinocephalus* and *Psathyrella* sp. were found most often. Additional fungus gnat species were observed to feed and oviposit on these fungi (Fig. 2C, D). We observed some fungus gnats feeding on secretions of the hymenophore surface (Fig. 2B) and found insect eggs on the fungi (Fig. 2D). However, these fungus gnats were physically larger than the *Phthinia* species and were not observed visiting flowers of *C. geminigibbus.*

Following 150 h of observation of flowers of *C. shanlinshiensis*, we observed three visits by gnats (*Exechia* sp.; Mycetophilidae). We caught two of these specimens as they exited the flowers, a female and a male. One entered a flower and remained there over 30 min (Fig. 3C). It struggled to escape and carried a dorsally deposited pollinarium on its thorax flying off immediately after escaping from the floral sinus. Our observations indicated that, on a daily basis, these gnats flew around orchid flowers from 11:00–13:00 and then again from 16:00–17:00. We caught a second female of an *Exechia* species carrying a pollinarium on its thorax (Fig. 3D) while it flew around these flowers. The thoracic depth of these specimens was significantly lower than the depth between the stigma and lip surface of the flower (U = 0.000, *P* < 0.01). There were few mushrooms in this site (*Laccaria* sp*.*, *Lacatrius volemus;* Table S1) and we did not see any insects feeding, laying eggs, or mating on these mushrooms near the orchid flowers. From 17:00 to 18:00, we observed some fungus gnats digging themselves down into the detritus and they did not reemerge after sunset. We found maggots in this detritus but could not see what they were consuming.

### Scent analyses

After deduction of compounds from the air control, we detected four compounds from two samples of *C. geminigibbus.* They consisted of β-Pinene, 1-Octanol, L-α-Terpineol and Longifolene. Four compounds were detected in flowers of *C. shanlinshiensis*, including Heptanal, β-Pinene, L-α-Terpineol and Longifolene. Three mushroom samples from the habitat of *C. geminigibbus* shared the typical mushroom scent of 1-Octen-3-ol, while the dominant compound of the *Mycena* sp. was linalool. Consistently, two mushroom samples from the habitat of *C. shanlinshiensis* produced two of the typical mushroom scents of 1-Octen-3-ol and 3-Octanone. One compound, 1-Octanol, was shared by the flowers of *C. geminigibbus* and *Mycena* sp., while longifolene was shared by the flowers of *C. geminigibbus* and *Suillus bovinus.* The compound L-α-Terpineol was detected in the flowers of *C. shanlinshiensis* and their co-occurring mushroom *Laccaria* sp. (Table 2).

**Table 2:** Relative abundance of compounds identified by GC–MS from head space samples of orchids *Corybas geminigibbus* and *C. shanlinshiensis*, and their sympatric mushrooms (*Suillus bovinus*, *Mycena* sp. and *Laccaria* sp.). *Suillus bovinus* and *Mycena* sp. were from the same habitat with *C. geminigibbus*, while *Laccaria* sp. came from the same habitat as *C. shanlinshiensis*. Compounds listed in order of increasing retention time after excluding compounds in the air control

Compounds	Retention time (min)	*Corybas geminigibbus*		*Suillus bovinus*		*Mycena* sp.	*Corybas shanlinshiensis*		*Laccaria* sp.	
Label of Sample		1	2	1	2	1	1	2	1	2
No. of flowers/mushroom bodies		12	12	6	6	6	6	6	6	6
Heptanal	8.49	-	-	-	-	-	50.90	-	-	-
β-Pinene	11.56	45.45	-	-	-	-	49.10	13.04	-	-
1-Octen-3-ol	11.78	-	-	100	89.66	4.43	-	-	22.06	9.69
3-Octanone	12.06	-	-	-	-	-	-	-	68.77	57.88
2-Octen-1-ol, (E)-	15.73	-	-	-	-	-	-	-	1.94	3.01
1-Octanol	15.88	-	54.46	-	-	3.73	-	-	1.87	6.24
Linalool	17.01	-	-	-	-	91.83	-	-	2.98	4.29
L-α-Terpineol	20.46	54.55	-	-	-	-	-	33.22	2.38	18.89
Longifolene	22.49	-	45.54	-	10.34	-	-	53.74	-	-

### Pollination treatments and fruit set

Bagged but unmanipulated flowers failed to set fruits in both species. Hand self- and hand cross-pollinated stigmas produced fruits (Table 3). There were significant differences among treatments for both species (LR Chisq = 39.796, df = 3, *P* < 0.001 for *C. geminigibbus*; LR Chisq = 26.624, df = 3, *P* < 0.001 for *C. shanlinshiensis*). In both species, fruit set produced by hand-manipulated cross-pollination and natural (insect-mediated) pollination was lower compared to hand self-pollination (all *P* < 0.001, Kruskal–Wallis test). The conversion rate of ovaries into fruits for flowers exposed to insects in the XBH population of *C. geminigibbus* was > 23% higher than in *C. shanlinshiensis.*

**Table 3:** Breeding systems of *Corybas geminigibbus* and *C. shanlinshiensis* in southwestern Yunnan. We only conducted breeding system experiment for *C. geminigibbus* at one site Xiangbaihe (XBH)

Treatments	Number of flowers	Number of fruits	Fruit set (%)
*Corybas geminigibbus*			
Bagged control	15	0	0
Hand self-pollination	10	9	90
Hand cross-pollination	10	4	40
Natural pollination (XBH)	68	21	30.8
*Corybas shanlinshiensis*			
Bagged control	15	0	0
Hand self-pollination	15	10	66.7
Hand cross-pollination	15	7	46.7
Natural pollination	110	8	7.27

## Discussion

### Neither species is an obvious brood site mimic

This is the first report of fungus-gnat pollination of *Corybas* species in situ in the Northern Hemisphere. Under natural conditions our observations show some parallels with those of Kuiter and Findlater-Smith [19]. First, as in Australia, our *Corybas* species were more likely to attract insects after morning hours. Second, different species of fungus gnats appeared to be pollinators of two different *Corybas* species. It’s possible that one of our orchid species may depend only on female gnats while the second may depend on males and females. Unfortunately, we could not obtain enough flower-visiting specimens to contrast, the role that sexual dimorphism may play in the efficiency of gnats as pollinia vectors as in Kuiter and Findlater-Smith [19]. However, as anticipated, differently sized flowers appeared to exploit differently sized pollinia vectors.

More important, the presence of male gnats on or in the flowers of one species, and the absence of eggs in the flowers of both species suggests we must reconsider and/or reinterpret previous theories of brood site mimesis as applied to the *Corybas* species in this study as reinterpreted previously by Kuiter and Findlater-Smith [19]. While there were 16 basidiomycete species in the *C. geminigibbus* site we can’t say that any of them were specific models for the putative mimic. In contrast, some members of the Neotropical genus *Dracula* appear to mimic the morphology and scents of some fungi identified to species within the ranges of these orchids [11, 12].

We also need to note that mushroom density and diversity in our *C. shanlinshiensis* site was far lower compared to the *C. geminigibbus* site. Furthermore, fruit set in *C. geminigibbus* in 2020 was higher compared to fruit set in *C. shanlinshiensis.* Are we looking at a classic example in which the reproductive success of a mimic species is more likely to increase if its flowering population is inundated and/or outnumbered by its models [29]? To answer this question properly, the same orchid and fleshy fungi populations must be monitored over additional seasons.

Consider the amount of time we spent observing these flowers of both species versus our infrequent sightings of gnats actually entering floral sinuses. Consider also the extended, two-week lifespan of such small flowers and their lack of edible rewards. Both traits are indicative of orchids with mimetic flowers [1, 2] but what mode or modes of mimesis did we find here?

### The fungus gnat pollination syndrome *versus* a shelter flower?

To explore this question, we must first note that fungus gnat pollination is not unique to the genus *Corybas* or to members of the Orchidaceae. While pollination by fungus gnats in the families Keroplatidae, Mycetophilidae and Sciaridae remain underexplored, it is also documented in 20 genera in eight families of unrelated angiosperms [14, 30]. Their flowers often share a deep and dark red pigmentation as observed in our two *Corybas* species. However, deep/dark red must not be interpreted specifically as the color of either food or brood site mimicry in orchids pollinated exclusively by fungus gnats. In fact, male mycetophilids attempt to copulate with the red labellum in the red and green flowers of *Pterostylis* (*Urochilus*) *orbiculatus* (D.L.Jones & M.A. Clem.) D.L. Jones & M.A. Clem. (syn. *Pterostylis sanguinea* D. L. Jones & M. A. Clem.) [31]. Therefore, while deep or dark red pigmentation may attract fungus gnats of both sexes it may also serve as a filter to exclude other insects from visiting the same flowers.

It may be more important to compare the flowers of orchids pollinated exclusively by fungus gnats with traits shared by fungus-gnat flora in other families. In these unrelated taxa dependent on fungus gnats, botanists were more likely to describe small, flat, actinomorphic flowers with exposed nectaries, with an androecium consists of more than one staminal filament all terminating in short anthers [8, 30, 32–35]. *Corybas* species differ sharply from this mode of presentation with their bilaterally symmetrical flowers forming domed floral chambers with spurs. Their pollinators must climb into the flowers to contact the rostellum and pollen-receptive lobes of the stigma. In the absence of nectar and gnat eggs in our *Corybas* flowers we suggest an alternative explanation why some female and/or male gnats enter these flowers at all. As some fungus gnat species enter the humus and leaf litter for shelter (see above) and then lay their eggs in decaying wood are we looking at flowers mimicking temporary shelters instead of a true brood site or even a food mimic? Dark colored shelter flowers have been described for male bees visiting flowers of *Serapias vomeracea* Briq. (Orchidoideae) [36] and “black” flowers in the *Oncocyclus* Section of genus *Iris* [37]. Obviously, this requires more work on behavior, life-span and egg-laying preferences of the Mycetophilidae. Moreover, Kelly and Gaskett [38] studied the floral reflectance of *C. cheesemanii*. They found that their flowers reflected strongly across all visible wavelengths, including UV, but mushrooms and surrounding leaf litter reflected red predominantly and no UV patterns suggesting that *C. cheesemanii* was not masquerading as a mushroom.

### What do scent analyses tell us?

Kelly and Gaskett [38] used the SPME GC–MS method to collect and analyze the floral scent of *C. cheesemanii*, but failed to detect any odours, although their noses detected a scent in their flowers. In the Neotropics *Dracula* species mimic the scent and sculptures of mushrooms in habitats and are pollinated by female drosophilids [11, 12, 39, 40]. The signature molecules, shared by *Dracula* species and model mushrooms include fungal volatiles, 1-octen-3-ol and 3-octanone. While these mushroom volatiles were produced by our mushrooms in situ, they were absent in our *Corybas* species so we cannot argue in favor of a generalist fungal mimesis [12, 39]. We do note that heptanal and terpineol groups are detected in many orchid flowers and unrelated angiosperms, with different pollinator systems [40]. In contrast, Longifolene, and β-Pinene are also components detected in the scents of conifer needles and old wood [40]. They may contribute to our shelter flower theory if they mimic some of the scents in forest detritus, in general, where adult gnats hide and/or search for mycelia. We remind the reader that our scent collections of both *Corybas* species were made using picked flowers uncontaminated by detritus. Furthermore, while 1-Octanol was a dominant molecule in one sample of *C. geminiggibus* it is not classified as a mushroom scent by Kaiser [40] or Raguso (personal communication).

We must note that linalool was the dominant component in the scent of one of the *Mycena* species and this is intriguing. While it was absent in both of our *Corybas* species it is common and often dominant in scents produced by both flowers and wood of dozens of unrelated angiosperms [40]. Could our *Mycena* sp. be mimicking a forest flower, or a fallen branch, or rotting stump?

### Self-compatibility without mechanical autogamy

Bagging and hand pollination experiments on both species showed that both are self-compatible but mechanical self-pollination (autogamy) did not occur. This is in direct contrast to hand-pollination experiments on *C. ecarinatus* in Thailand in which hand self-pollination failed to produce fruits but hand cross-pollination had a 30% rate of success [22]. Reports of self-incompatibility in the Orchidaceae are uncommon and understudied [1, 20]. It is tempting to suggest that both of our species showed a trend towards zoophilous self-pollination as fruit set following hand self-pollination was higher compared to hand cross-pollination. However, this interpretation lacks the definitive analysis of seed set. We have found that it is common for orchid species in several lineages to produce fruit following self-pollination but, when this occurs, capsule contents show a higher frequency of empty seeds and underdeveloped embryos [25, 26, 41]. Comparative rates of seed development following hand-pollination must be tested before future conservation programs for *Corybas* species in Asia are enacted as they will surely involve limited population rescues followed by in vitro programs of hand-pollinations.

## Supplementary Information

**Additional file 1:** **Table S1.** Mushroom species list at three study sites in southwestern Yunnan, China.

## Data Availability

All data generated or analysed during this study are included in this published article.
