# Supplementary material for: Observations on the pollination and breeding systems of two Corybas species (Diurideae; Orchidaceae) by fungus gnats (Mycetophilidae) in southwestern Yunnan, China
Source: BMC Plant Biol. 2022 Sep 2;22:426. doi: 10.1186/s12870-022-03816-1 (PMC9438300; doi:10.1186/s12870-022-03816-1)
Supplement: Supplementary file 1 — Additional file 1: Table S1. Mushroom species list at three study sites in southwestern Yunnan, China. [file 12870_2022_3816_MOESM1_ESM.docx]

**Table S1.** Mushroom species list at three study sites in southwestern Yunnan, China

| Site | Orchid species | No. mushroom species | Mushroom species |
| --- | --- | --- | --- |
| XBH | *Corybas geminigibbus* | 14 | *Cortinarius alboviolaceus*, *Cortinarius* sp., *Entoloma anatinum*, *Entoloma caesiellum*, *Entoloma pyrospilum*, *Lactarius lignicola*, *Lacatrius gracilis*, *Lactarius subindigo*, *Marasmius haematocephalus*, *Marasmiellus* sp., *Mycena filopes*, *Psathyrella* sp., *Ramaria* sp., *Russula* sp. |
| MBZ | *Corybas geminigibbus* | 4 | *Mycena* sp., *Psathyrella* sp., *Strobilomyces echinocephalus*, *Suillus bovinus* |
| HQZ | *Corybas shanlinshiensis* | 2 | *Lacatrius volemus*, *Laccaria* sp. |
